# FAMILIES AND DEMENTIA: ESTIMATES AND EXPOSURES

**DOI:** 10.1093/geroni/igad104.0346

**Published:** 2023-12-21

**Authors:** Esther Friedman, Vicki Freedman, Sarah Patterson

**Affiliations:** University of Michigan, Ann Arbor, Michigan, United States; University of Michigan, Ann Arbor, Michigan, United States; University of Michigan, Ann Arbor, Michigan, United States

## Abstract

Roughly 10 to 20% of older adults in the U.S. have dementia, which could have implications for their families and household members, who are likely to be called upon to provide care. Yet, it is not clear how many families and households include someone with dementia. This study provides national level estimates of families and households with an older adult with possible dementia using the 2017 Panel Study of Income Dynamics (PSID). Consistent with prior work, we find that 21.5% of older adults 65+ have possible dementia. Moreover, more than a quarter (26.3%) of households with an adult aged 65+ include an older adult with dementia, and 37.0% of extended family networks (with at least one adult aged 65+) have at least one older adult with dementia. Individual-level stratification of dementia by racial-ethnic group and socioeconomic status translates directly to household- and family-level patterns. For instance, households and families with an older adult who is either Black or Hispanic or does not hold a college degree are more likely to include at least one person with possible dementia. These findings have implications for the extent to which American families and households include someone with dementia, which comes with a risk of serving as a caregiver as well as possible implications for health and well-being. Our findings also establish important baseline estimates that can be measured against future estimates, for instance those post COVID-19.

